# Incontinence and Erectile Dysfunction Following Radical Prostatectomy: A Review

**DOI:** 10.1100/tsw.2005.94

**Published:** 2005-09-13

**Authors:** Gerasimos Alivizatos, Andreas Skolarikos

**Affiliations:** Athens Medical School, Second Department of Urology, Sismanoglio Hospital, Greece

**Keywords:** incontinence, erectile dysfunction, radical prostatectomy, incidence, evaluation, pathophysiology, treatment, quality of life

## Abstract

Radical prostatectomy remains the treatment of choice for localized prostate cancer in age-appropriate and health-appropriate men. Although cancer control is the most important aspect of a radical prostatectomy, minimization of postoperative morbidity, especially urinary incontinence and erectile dysfunction, is becoming a greater concern. We reviewed recent data available on Medline regarding the incidence, pathophysiology, evaluation, and treatment of incontinence and sexual dysfunction after radical prostatectomy. Health-related quality of life issues have been specifically addressed. Although low incidences of incontinence and erectile dysfunction after radical prostatectomy have been reported in the hands of experienced surgeons, the literature review revealed a great variety, with incontinence rates ranging from 0.3–65.6% and potency rates ranging from 11–87%. Several factors contribute to this wide difference, the most important being the application of a meticulous surgical technique. General and cancer-specific health-related quality of life is not being affected after radical prostatectomy. The incidence of incontinence and erectile dysfunction is higher after radical prostatectomy when compared to the incidence observed when other therapies for localized prostate cancer are applied. However, the majority of the patients undergoing radical prostatectomy would vote for the operation again. Today, avoidance of major complications after radical prostatectomy depends mostly on a high-quality surgical technique. When incontinence or erectile dysfunction persists after radical prostatectomy, the majority of the treated patients can be managed effectively by various methods.

## INTRODUCTION

Prostate cancer is the most common male cancer in the Western world and the second most common cause of cancer death[1]. The introduction of prostate specific antigen (PSA) in the late 1980s changed the diagnosis of this disease dramatically and, as a result, radical prostatectomy became one of the most commonly performed surgical procedures[2]. Younger patients in their fifth and sixth decades are now being operated on and despite the evolution of the surgical technique described by Walsh[3], two major side effects, incontinence and erectile dysfunction, still affect the quality of life (QOL) of these patients.

The purpose of this review is to present recent data dealing with these major complications of radical prostatectomy (RP) and the way they affect QOL, hoping that the information will help the undecided practicing urologists — those who work in large academic hospitals, but also those who operate in smaller community or private centers — to find which is the optimal treatment for their own patients within their own settings.

## URINARY INCONTINENCE AFTER RADICAL PROSTATECTOMY

### Incidence and Quality of Life

Historically, the incidence of incontinence following RP has been quoted as being approximately 5%. However, recent studies[4,5,6,7,8,9,10,11,12,13,14,15,16,17,18,19,20,21,22] indicate that perhaps the true incontinence rate is higher, ranging from 0.3–65.6% (Table 1). There are several factors that contribute to this wide difference. Lack of consensus regarding the definition of incontinence, data derived from single vs. multi-institutional studies, and patient vs. physician-reported data are important elements. Patient age and surgical experience also influence the outcome. Some studies also included patients who were incontinent preoperatively**.** Additionally, because incontinence rates are noted to decrease over time from surgery, the duration of follow-up is also important[4,5,6,7,8,9,10,11, 12,13,14,15,16,17,18,19,20,21,22].

General and cancer-specific health-related quality of life scores (HRQOL) do not differ among patients with prostate cancer undergoing RP and age-matched healthy individuals[23] or patients with prostate cancer who are subjected to watchful waiting[24]. However, urinary incontinence and sexual domain scores are lower in the RP patients, indicating a poor health state[23,24].

A number of recent studies have compared HRQOL issues across standard therapies for localized prostate cancer[23,25,26,27,28] (Tables 2 and 3). These studies have depicted that even after adjustment for patient age, follow-up time, and comorbidity, external beam radiation therapy (EBRT) patients and brachytherapy patients tended to have poorer general health function and bowel function compared to RP patients. However, men receiving radiation therapy are less likely than men undergoing RP to be incontinent and to suffer from sexual dysfunction[12,23,25,26,27,28]. Although urinary incontinence is perhaps the patient's most feared postoperative complication due to its severe social implications, most patients would select RP as a treatment again[7,8,9].

### Pathophysiology and Evaluation

There are various types of incontinence after RP such as stress, urge, overflow, and total incontinence and various causative reasons such as sphincteric insufficiency, storage insufficiency, detrusor hypotonicity, and outlet obstruction. Total incontinence is rare and always the result of damage to the sphincteric mechanism. Urge incontinence occurs during uninhibited detrusor activity due to decreased compliance, detrusor instability, or a combination of both. Stress incontinence occurs in varying degrees after RP and is attributed to post-treatment sphincteric insufficiency, pre-existing or newly developed bladder storage insufficiency, or rarely, to both. Finally, overflow incontinence results from an obstructing urethrovesical anastomotic stricture[29]. Sphincteric dysfunction appears to be the main and more frequent cause of post-RP incontinence, comprising approximately 32.5–92% of the cases. Bladder dysfunction is rarely the sole cause (3–40% of the cases), but it may often coexist with sphincteric dysfunction (23–52% of cases)[30,31].

**TABLE 1**
**Post-RP Incontinence Rates**

| *Author* | *Technique* | *No. of Patients* | *Follow-Up Period (Months)* | *Rate of Incontinence (%)* |
|---|---|---|---|---|
| Bishoff et al.[4] | Retropubic and perineal | 1200 | 12 | 47 |
| Horie et al.[5] | Retropubic | 104 | 12 | 22 |
| Kleinhans et al.[6] | Retropubic | 44 | 12 | 0.3 |
| Walsh et al.[7] | Retropubic | 62 | 18 | 7 |
| Fontaine et al.[8] | Various | 116 | 54 | 14.4 |
| Kao et al.[9]* | Various | 1069 | >6 | 65.6 |
| Artibani et al.[10] | Retropubic | 50 | 12 | 36 |
| Lepor and Kaci[11] | Retropubic | 500 | 12 | 7.9 |
| Penson et al.[12] | Various | 1288 | 60 | 14 |
| Bollens et al.[13] | Laparoscopic | 50 | 6 | 15 |
| Guillonneau et al.[14] | Transperitoneal laparoscopic | 550 | 12 | 17.7 |
| Salomon et al.[15] | Transperitoneal laparoscopic | 300 | 12 | 10 |
| Dahl et al.[16] | Transperitoneal laparoscopic | 70 | 3 | 15 |
| Rassweiler et al.[17] | Transperitoneal laparoscopic | 500 | 12 | 5 |
| Stolzenburg et al.[18] | Extraperitoneal laparoscopic | 300 | 12 | 10.4 |
| Eden et al.[19] | Transperitoneal laparoscopic | 100 | 12 | 10 |
| Eden et al.[19] | Extrperitoneal laparoscopic | 100 | 12 | 4 |
| Rozet et al.[20] | Extrperitoneal laparoscopic | 100 | 12 | 14 |
| Patel et al.[21] | Robotic | 200 | 12 | 2 |
| Menon et al.[22] | Robotic | 565 | 6 | 4 |

*This study refers to patients who underwent RP between 1962 and 1997.

In order to evaluate incontinence after RP, a careful history must be obtained. The type of incontinence, its onset, the duration and the severity, its improvement or worsening during follow-up, as well as the voiding diary the patient keeps, must be appropriately registered. Physical examination can rule out bladder overdistension and define if normal sacral motor, sensory, and reflex neurological function is present[29]. Urethral strictures or urethrovesical anastomotic contractures can be recognized easily during cystoscopy. When urge incontinence predominates, urinalysis and urine culture should be performed to rule out urinary tract infection. A serum PSA value can exclude persistent or recurrent prostate cancer as the cause of overflow incontinence due to obstruction caused by local recurrence[29]. Simple urodynamic studies should be performed postoperatively, especially when incontinence persists. Flowmetry, filling, and voiding cystometry, and sometimes pressure-flow studies under fluoroscopy, can easily demonstrate outlet obstruction, unstable detrusor muscle, low or high bladder compliance, and finally intrinsic sphincteric deficiency[29].

**TABLE 2**
**Generic HRQOL Studies Demonstrating Differences in HRQOL Across Therapies**

| Author | No. of Patients | HRQOL Instrument* | Follow-Up | *Mean HRQOL Scores by Therapy* | | | |
|---|---|---|---|---|---|---|---|
| | | | | RP | EBRT | Brachytherapy | Watchful Waiting |
| Davis et al.[26] | 528 | SF 36 | 5 years | | | | |
| | | Physical function | | 83.3 | 74.3 | 80.8 | — |
| | | Role limitation | | 74.6 | 58.6 | 69.1 | — |
| | | Bodily pain | | 82.9 | 78.0 | 78.8 | — |
| | | General health | | 70.9 | 63.9 | 66.3 | — |
| | | Vitality | | 67.8 | 65.5 | 62.5 | — |
| Litwin et al.[27] | 452 | SF 36 | 2 years | | | | |
| | | Mental health | | 85 | 75 | — | 81 |
| | | Role limitation | | 94 | 81 | — | 86 |
| | | Vitality | | 73 | 61 | — | 66 |
| | | Social function | | 100 | 86 | — | 89 |
| Bacon et al.[28] | 842 | SF 36 | 5 years | | | | |
| | | Physical function | | 90 | 83 | 90 | 79 |
| | | Role limitation | | 86 | 72 | 79 | 85 |
| | | Bodily pain | | 85 | 79 | 81 | 81 |
| | | General health | | 80 | 74 | 78 | 71 |
| | | Vitality | | 71 | 64 | 66 | 68 |
| | | Social function | | 92 | 87 | 92 | 87 |
| | | Emotional function | | 90 | 82 | 86 | 90 |
| | | Mental health | | 84 | 81 | 84 | 83 |
| | | Physical component | | 52 | 49 | 51 | 49 |
| | | Mental component | | 55 | 53 | 54 | 55 |

*Higher scores on the Sort Form 36 (SF 36) represent better HRQOL.

## Prevention and Treatment

The anatomic dissection that Walsh first described permitted surgeons to maximize urethral length and perform a tension-free, leak-free, mucosa-to-mucosa vesicourethral anastomosis[3]. It appears as though careful apical dissection, with care not to injure the distal urethral sphincter, irrespective to the preservation or not of the neurovascular bundles, is the most important factor in the maintenance of continence[3,32,33]. Bladder neck preservation does not seem to have a major impact on continence rate, but may decrease anastomotic strictures rates and contributes substantially to continence’s earlier recovery[34,35,36]. Preservation of the puboprostatic ligaments during the RP may improve continence by maintaining continuity of the ligamentous attachments to the urethra and the sphincter[33]. Preserving the external striated urethral sphincter, its innervations, and its fascial attachments leads to a shorter period of incontinence[32,33].

**TABLE 3**
**Prostate Cancer-Specific HRQOL Studies Demonstrating Differences in HRQOL Across Therapies**

| Author | No. of Patients | HRQOL Instrument* | Follow -Up | *Mean HRQOL Scores by Therapy* | | | |
|---|---|---|---|---|---|---|---|
| | | | | RP | EBRT | Brachytherapy | Watchful Waiting |
| Wei et al.[23] | 1014 | EPIC summary measures | 4 years | | | | |
| | | Urinary irritative | | 89.6 | 84.2 | 71.5 | — |
| | | Urinary incontinence | | 77.5 | 92.8 | 82.1 | — |
| | | Bowel | | 93.2 | 85.2 | 76.0 | — |
| | | Sexual | | 33.9 | 38.8 | 26.9 | — |
| | | Hormonal | | 90.9 | 87.2 | 83.7 | — |
| Wei et al.[23] | 1014 | FACT P | 4 years | 36.9 | 36.4 | 32.4 | — |
| Potosky et al.[25] | 1591 | PCOS** | 2 years | | | | |
| | | Incontinence bother | | 11.2% | 2.3% | — | — |
| | | Bowel bother | | 3.3% | 8.4% | — | — |
| | | Sexual bother, ages 55–59 years | | 59.4% | 25.3% | — | — |
| | | Sexual bother, ages 60–74 years | | 53.2% | 46.1% | — | — |
| Davis et al.[26] | 528 | PCI Domains | 5 years | | | | |
| | | Bowel function | | 85.5 | 76.8 | 82.5 | — |
| | | Bowel bother | | 83.0 | 71.8 | 79.3 | — |
| | | Sexual function | | 17.9 | 26 | 32.2 | — |
| | | Sexual bother | | 25.2 | 40.0 | 40.4 | — |
| | | Urinary function | | 68.4 | 86.4 | 86.8 | — |
| | | Urinary bother | | 73.9 | 82.6 | 76.8 | — |
| Bacon et al.[28] | 752 | PCI domains | 5 years | | | | |
| | | Bowel function | | 86 | 81 | 80 | 91 |
| | | Bowel bother | | 86 | 78 | 72 | 89 |
| | | Sexual function | | 26 | 34 | 36 | 54 |
| | | Sexual bother | | 43 | 51 | 54 | 74 |
| | | Urinary function | | 76 | 89 | 87 | 93 |
| | | Urinary bother | | 82 | 83 | 75 | 89 |

* Higher scores on the Prostate Cancer Index (PCI), Expanded Prostate Cancer Index: Composite (EPIC), and Functional Assessment of Cancer Therapy Prostate Module (FACT-P) represent better HRQOL.

** Prostate Cancer Outcomes Study Questionnaire (PCOS) reports the percent of patients being bothered for each domain.

After urge incontinence and overflow incontinence have been ruled out or treated, the management of incontinence depends on the time period from surgery. Most authors believe it is best to wait 9–12

months before surgical intervention. During this period, conservative treatment can be applied to these patients. Voiding at lower bladder volumes and pharmacological therapy using alpha agonists may help patients with mild stress incontinence[29]. Other conservative measures include pelvic floor muscle training, biofeedback, electrical stimulation using a rectal electrode, transcutaneous electrical nerve stimulation, or a combination of these methods. The value of these approaches remains uncertain. There may be some benefit of offering pelvic floor muscle training with biofeedback early in the postoperative period immediately following removal of the catheter, as it may promote an earlier return to continence[37].

When the decision is made to proceed with surgical treatment, several options exist: injection therapy, slings, and an artificial urinary sphincter (AUS). Injectable therapy for postprostatectomy incontinence has been notoriously associated with low success rates of 20–35%[38]. Issues related to the success of injectable therapy include patient selection, type of injected material, volume of each injection, and number of injections. Patient selection remains the most important determinant, with low success rates observed in patients with severe postprostatectomy incontinence[38]. Data regarding postprostatectomy sling procedures are preliminary and will require long-term confirmation[39,40,41,42, 43,44,45,46,47]. It seems that some patients will eventually benefit from the procedure, which is not free of complications, however (Table 4). The AUS is considered by many to be the gold standard for treating postprostatectomy incontinence resulting in continence rates as high as 79–96%[33,48,49]. In a recent study, 71 patients who underwent RP, had an AUS inserted, and were followed for a mean period of 7.7 years, experienced a high rate of continence and satisfaction[48]. Surgical revision was needed in 29% of them due to mechanical failure, erosion, or infection. Interestingly, patient dissatisfaction with the procedure was not correlated with the number of surgical revisions, but with the number of pads used.

## IMPOTENCE AFTER RADICAL PROSTATECTOMY

### Incidence and Quality of Life

Prior to the introduction of nerve-sparing techniques, impotence after radical prostatic surgery was the rule. Walsh's studies using a nerve-sparing technique reported potency rates as high as 69 and 86% if one or both neurovascular bundles were spared, respectively[3]. Other studies, however, have been less optimistic, showing potency rates ranging from 11–87% (Table 5). This variance appears to be surgeon-dependent, but it may also reflect the nonuniformity in data collection. The criteria of either a positive erectile response or sexual satisfaction are not applied universally. Variables include the qualitative difference between a partial and a full erection, the percentage of rigid erections/attempts, and the duration of vaginal intercourse. Moreover, the most important factors that appear to be associated with a higher rate of postoperative potency include younger age, localized cancer, preservation of both neurovascular bundles, and normal preoperative sexual activity[7,50,51,52,53,54,55,56].

The majority of patients who undergo RP have a moderately or severely affected QOL because of their postoperative erectile dysfunction, when the later is investigated[23,57,58]. Erectile dysfunction is more common among patients undergoing RP as compared to those subjected to watchful waiting, EBRT, or brachytherapy[23,24,25,26,27,28]. However, there was no statistically significant difference among various groups when distress from erectile dysfunction, decreased frequency of intercourse, or compromised sexuality was examined. This is probably why patients are not willing to spend a lot of their remaining life to achieve perfect sexual function and the majority of them would vote for surgery again, indicating that postoperative erectile dysfunction is not an independent predictor for global QOL[23,24,25,26,27,28,29,57,58].

**TABLE 4**
**Male Slings in Management of Postprostatectomy Incontinence**

| *Author* | *No. of Patients* | *Mean Follow-Up (Months)* | *Sling Material* | *Cure (%)* | *Improved (%)* | *Complications* |
|---|---|---|---|---|---|---|
| Schaeffer et al.[39] | 64 | 22.4 | Cooley soft graft material or polyethelene terephthalate | 56 | 5 | Revision, 27%; erosion, 6%; infection, 3%; perineal discomfort, 52% |
| Madjar et al.[40] | 16 (8 RRP*) | 12.2 | Gelatin-coated polyethelene terephthalate or cadaveric fascia lata | 75 | 25 | Perineal discomfort, 18.7% |
| Defidio et al.[41] | 15 | 2–9 | Cadaveric fascia lata | 86.6 | 6.6 | Urinary infection, 20% |
| Comiter et al.[42] | 67 | 12 | Prolene | 76 | 19 | No erosion, infection |
| Migliari[43] | 9 | 14 | Polypropelene | 55.5 | 22.2 | Perineal/genital discomfort, 55.5% |
| John et al.[44] | 16 | 14 | Polypropylene | 69 | 6 | |
| Rajpurkar et al.[45] | 46 | 24 | Polyester coated with porcine dermis | 37 | 37 | Perineal/buttock pain, 4.3%; infection, 2.1% |
| Castle et al.[46] | 38 | 18 | Polyester coated with porcine dermis | 15.8 | 39.5 | Erosion, 2.6%; infection, 7.8% |
| Stern et al.[47] | 71 | 48 | Tetrafluoroethylene bolsters | 36 | 68 | Perineal discomfort, 33%; sling removal, 8.4% |

*RRP: RP patients.

## Pathophysiology

Before Walsh's nerve-sparing technique was described[3], the surgical techniques of RP were less anatomical and relied often on blind and blunt dissection. It appears that neurovascular bundles were damaged during apical dissection and transection of the urethra, as well as during the separation of the prostate from the rectum or division of the lateral pedicle.

Despite nerve damage being the principal issue of post-RP impotence in non-nerve-sparing RP, there are some data suggesting that there may be a vasculogenic component as well[59,60]. Apart from the arteries of the neurovascular bundles, injury of the accessory pudendal arteries that are invariably found during the operation and the division of arterial branches running beneath the anterior capsule of the prostate that are visible following division of the dorsal complex are also responsible for erectile dysfunction postprostatectomy[59]. The postoperative combination of reduced penile arterial inflow and excessive venous outflow due to the apoptosis-induced damage of the venous occlusive mechanism, lead to reduced oxygen transport and increased production of transforming growth factor beta[60,61]. This subsequently causes significant tissue damage, i.e., increased corporeal fibrosis, which is the cause for the penile hemodynamic abnormality[60,61].

**TABLE 5**
**Prevalence of Erectile Dysfunction Following RP**

| *Author* | *No. of Patients* | *Follow-Up (Months)* | *Potency Rates (%)* | | | |
|---|---|---|---|---|---|---|
| | | | BNS* | UNS* | NNS* | Total |
| Quinlan et al.[50] | 503 | 12 | 76 | 60 | 0 | — |
| McCammon et al.[51] | 203 | 42 | 45.2 | 30.5 | 16.7 | — |
| Geary et al.[52] | 459 | 12 | 31.9 | 13.3 | 11 | — |
| Penson et al.[12] | 1288 | 18 | 44 | 41.4 | 34.4 | — |
| Catalona and Basler[32] | 295 | 12 | 63 | 41 | — | — |
| Leandri et al.[53] | 106 | 12 | — | — | — | 71 |
| Drago et al.[54] | 151 | — | — | — | — | 66 |
| Walsh et al.[7] | 64 | 18 | — | — | — | 86 |
| Kao et al.[9] | 1069 | >6 | — | — | — | 11.6 |
| Guillonneau et al.[14] | 40 | 6 | — | — | — | 45 |
| Bollens. et al.[13] | 6 | 6 | 67 | — | — | |
| Salomon et al.[15] | 143 | 12 | 87.5 | 50 | 30 | 56 |
| Su et al.[55] | 177 | 12 | 76 | — | — | 48 |
| Eden et al.[19] | 100 | 12 | — | — | — | 61 |
| Eden et al.[19] | 100 | 12 | — | — | — | 82 |
| Roumeguere et al.[56] | 33 | 12 | 54.5 | — | — | — |
| Roumeguere et al.[56] | 26 | 12 | 65.3 | — | — | — |
| Rozet et al.[20] | 100 | 6 | 64 | 43 | — | — |
| Menon et al.[22] | 565 | 6 | — | — | — | 64% |

* BNS: bilateral nerve-sparing technique; UNS: unilateral nerve-sparing technique; NNS: non-nerve-sparing technique.

Patients undergoing nerve-sparing RP often experience impairment of erections in the early postoperative period. This has been related to the development of neuropraxia, which is believed to be caused by some damage to the cavernosal nerves that inevitably occurs during the excision of the prostate, even in the hands of most experienced surgeons. Controversy exists regarding whether the potency rates are significantly different between bilateral vs. unilateral nerve-sparing RPs. A large community-based study failed to show a significant difference in the recovery of function in bilateral vs. unilateral nerve-sparing procedure[12]**.** Finally, it is important to remember that patient age, psychogenic factors, pre-existing potency status, coexisting comorbid conditions, and the time interval from surgery may contribute to erectile dysfunction[59].

## Prevention and Treatment

A meticulous, anatomical, nerve-sparing technique constitutes the cornerstone of prophylaxis from erectile dysfunction following RP. The better understanding of the pathophysiology of postprostatectomy erectile dysfunction including the concept of tissue damage induced by poor corporeal oxygenation paved the way to the application of oral phosphodiesterase-5 inhibitors (PDE5-I) or intracorporeal injections of alprostadil early after the operation. The continuous use of these agents postoperatively improve penile hemodynamic and contribute to earlier return of spontaneous erections[62,63,64].

Because the natural recovery of erectile function has been reported to take as long as 2 years[65], it is possible that the erectile rehabilitation may simply bring forward the natural healing time of potency rather than saving patients from permanent erectile failure. Larger randomized trials with at least 2 years of follow-up are required before a definitive conclusion can be drawn on the true efficacy of rehabilitative sexual therapy.

Cavernous nerve reconstruction and neuroprotection are the future management avenues in preventing erectile dysfunction following RP. Unilateral or bilateral autologous sural nerve grafting may result in achieving spontaneous erections sufficient for intercourse postoperatively[66,67]. Neuroprotective/neurotrophic interventions have also rapidly gained attention as an approach to promote or restore nerve function following RP. Multiple factors have been described as potentially active neurotrophic effectors in the penis. These include growth hormone, neurturin, immunophilins, sonic hedgehog protein, vascular endothelial factor, prostaglandin E1, brain-derived neurotrophic factor, and insulin-like growth factor. Delivery methods for neurotrophic factors have combined gene therapy, tissue engineering, and tissue reconstitution techniques. The fields of cavernous nerve reconstruction and of therapeutic neurogenesis are at the earliest development stages and they still need to undergo the test of time[60].

Most patients with erectile dysfunction following RP respond well to standard treatments. The field of erectile dysfunction was revolutionized in early 1998 with the introduction of sildenafil. An intact neurological mechanism must be present in order for patients to benefit by this drug. PDE5-Is obtain the best results following RP in young patients (those less than 60 years of age having the best response), in patients treated with a bilateral nerve-sparing procedure, and in patients who show some degree of spontaneous postoperative erectile function. Typically, the response to PDE5-I has been shown to improve as time passes after the procedure. Response rates of 15–80% are seen from 12–24 months postoperatively[68,69,70].

Patients either not responding to or who cannot use PDE5-I are typical candidates for second-line pharmacological treatment that currently includes the intraurethral and the intracorporeal administration of alprostadil or other vasoactive agents. Effectiveness of intraurethral alprostadil in general is in the range of 30–55% in providing an erection that can be used for intercourse. Its combination with oral sildenafil may salvage a significant proportion of sildenafil failures[71]. Intracorporeal injections with papaverine, phentolamine, and prostaglandin El, alone or in combination, are currently used effectively in the treatment of erectile dysfunction. Response rates as high as 90% have been reported[62,72].

Vacuum erection devices and especially penile prostheses provide a predictable and reliable outcome. Satisfaction rate among patients and partners with the use of penile prostheses is the highest of all among the various treatment modalities for erectile dysfunction, the rate being as high as 85%[73]. Penile prostheses may be the last resort for the post-RP impotent man after oral or intracavernous therapy has failed.

## CONCLUSIONS

Today, the various major complications after RP depend mostly on a meticulous surgical technique that is acquired after a long learning curve and can be minimized in centers where large numbers of this procedure are performed.

Post-RP incontinence is usually a minor problem and, therefore, does not have a great impact on the QOL of the treated patients. When present, its treatment (conservative or surgical) is not always successful.

Impotence affects the surgical patients more frequently, disturbs their QOL when present, depends mainly on the preservation of the neurovascular bundles, and can be managed effectively by oral medication, by intracavernous injections, and with the help of penile prostheses.
